# Enzymolytic soybean meal—impact on growth performance, nutrient digestibility, antioxidative capacity, and intestinal health of weaned piglets

**DOI:** 10.3389/fvets.2024.1381823

**Published:** 2024-03-22

**Authors:** Kai Tan, Zhiyao Bian, Huiqing Liang, Wenyue Hu, Miao Xia, Shuaijuan Han, Baojiang Chen

**Affiliations:** College of Animal Science and Technology, Hebei Agricultural University, Baoding, China

**Keywords:** antioxidative capacity, enzymolytic soybean meal, growth performance, intestinal health, piglets

## Abstract

Enzymolytic soybean meal (ESBM) enriches free amino acids and small peptides, while mitigating anti-nutritional factors. Substituting soybean meal with ESBM enhances animal performance, though optimal piglet dietary supplementation levels vary. The present study aimed to assess the impact of ESBM on the growth performance, nutrient digestibility, antioxidative capacity and intestinal health of weaned piglets. A total of 120 piglets (initial body weight, 7.0 ± 0.4 kg) were randomly allocated into 4 dietary groups, each comprising 5 replicates with 6 piglets per replicate. The control group received the basal diet, while the experimental groups were fed diets containing 2, 4% or 8% ESBM as a replacement for soybean meal over 28 days. Compared with the control group, piglets supplemented with 4% ESBM exhibited a significant increase (*p* < 0.05) in average daily gain and the apparent total tract digestibility of dry matter, ether extract and gross energy (*p* < 0.05), alongside a notable decrease (*p* < 0.05) in diarrhea incidence. Fed ESBM linearly increased (*p* < 0.05) the villus height in the ileum of piglets. The levels of superoxide dismutase and total antioxidant capacity in serum of piglets increased (*p* < 0.05) in the 2 and 4% ESBM groups, while diamine oxidase content decreased (*p* < 0.05) in the 4 and 8% ESBM group. ESBM inclusion also upregulated (*p* < 0.05) the expression of superoxide dismutase 1 (*SOD-1*), Catalase (*CAT*) and *claudin-1* mRNA. In terms of cecal fermentation characteristics, ESBM supplementation resulted in a increase (*p* < 0.05) in valerate content and a linear rise (*p* < 0.05) in propionate, butyrate, and total short-chain fatty acids levels, accompanied by a decrease (*p* < 0.05) in the concentrations of tryptamine and NH_3_ in cecal digesta. ESBM had no discernible effect on cecal microbial composition. In summary, substitution of soybean meal with ESBM effectively improved the growth performance of piglets by enhancing nutrient digestibility, antioxidant capacity, intestinal barrier and cecal microbial fermentation characteristics, with the optimal replacement level identified at 4%.

## 1 Introduction

Weaning stress often precipitates a myriad of challenges in piglets, including diminished feed intake, increased frequency of diarrhea, and compromised growth performance. This stress also sustained impairment of intestinal barrier function and oxidative damage, significantly impeding piglet growth (1–3). Moreover, the abrupt transition of piglets from easily digestible milk to solid diets rich in complex proteins exacerbates these challenges, potentially leading to severe consequences, such as diarrhea or mortality (4). While antibiotics have historically been utilized to enhance animal production, their indiscriminate and excessive use has raised concerns regarding antibiotic residues in animal products and the proliferation of antibiotic-resistant strains, posing substantial public health risks (5–7). Hence, the selection of easily digestible and premium-quality feed ingredients is paramount to mitigating these challenges and ensuring optimal piglet health and growth.

Soybean meal (SBM), a byproduct of soybean oil processing, serves as a primary plant protein source in swine diets. However, SBM harbors numerous antinutritional factors, including β-globulin, phytate or trypsin inhibitor, and nondigestible oligosaccharides like raffinose and stachyose. These components elicit gastrointestinal hypersensitive immune responses, leading to diarrhea and detrimentally impacting the intestinal health and growth of weaned piglets (8–10). Consequently, mitigating the concentration of antinutritional factors in SBM to enhance its efficacy has emerged as a key research focus. Enzymatic hydrolysis represents a widely accepted and safe method to reduce protein allergenicity by altering the structure and conformation of soybean protein, thereby diminishing sensitization and enhancing biochemical function (11). Enzymolytic soybean meal (ESBM), poised as a high-quality protein source replacing SBM in diets of piglets, has a higher proportion of short peptides and lower levels of antinutritional factors compared to SBM, thereby enhancing growth performance and nutrient digestibility (4, 12). Despite these advancements, limited studies have evaluated the effects of ESBM on antioxidant capacity and intestinal health in weaned piglets, while consensus regarding the optimal ESBM replacement level for SBM remains elusive. Therefore, this experiment seeks to assess the impacts of varying dietary replacement levels of SBM with ESBM on growth performance, nutrient digestibility, antioxidant capacity, intestinal health, and cecal fermentation characteristics of weaned piglets.

## 2 Materials and methods

### 2.1 SBM and ESBM

The ESBM was produced by using alkaline protease (200,000 U/g) and neutral protease (50,000 U/g), which was provided by Qinhuangdao Qihao Biotechnology Co., Ltd. (Qinhuangdao, China). The levels of free and hydrolyzed amino acids, oligosaccharides, and antinutritional factors in SBM and ESBM were analyzed. The concentrations of small peptide and free amino acids were higher, while the levels of raffinose, stachyose, sucrose, maltose, glycinin and β-Conglycinin were lower in ESBM than SBM (Tables 1, 2).

**Table 1:** Contents of hydrolyzed amino acids and free amino acids in SBM and ESBM (air-dry basis, %).

Hydrolyzed amino acid	Soybean meal	Enzymolytic soybean meal	Free amino acid	Soybean meal	Enzymolytic soybean meal
Aspartate	4.80	4.78	Aspartate	0.05	0.15
Glutamic acid	8.23	8.27	Glutamic acid	0.14	0.47
Serine	2.12	2.08	Serine	0.03	0.24
Arginine	3.29	3.05	Arginine	0.12	0.17
Glycine	1.96	1.95	Glycine	0.02	0.11
Threonine	1.70	1.72	Threonine	0.03	0.16
Proline	2.33	2.40	Proline	0.13	0.11
Alanine	1.92	2.01	Alanine	0.05	0.26
Valine	2.05	2.06	Valine	0.06	0.14
Methionine	0.34	0.37	Methionine	0	0.04
Cystine	0.53	0.36	Cystine	0	0.05
Isoleucine	1.93	2.05	Isoleucine	0.04	0.07
Leucine	3.40	3.41	Leucine	0.02	0.29
Phenylalanine	2.21	2.22	Phenylalanine	0.01	0.14
Histidine	1.02	1.01	Histidine	0.02	0.03
Lysine	2.72	2.7	Lysine	0.03	0.12
Tyrosine	1.41	1.36	Tyrosine	0.43	0.64
Total amino acid	41.95	41.8	Total amino acid	1.17	3.20

**Table 2:** Biochemical characteristics of SBM and ESBM.

Item	Soybean meal	Enzymolytic soybean meal
Raffinose, %	1.14	0.06
Stachyose, %	2.23	0.11
Sucrose, %	3.49	0.46
Maltose, %	0.45	0.01
Glycinin, mg/g	105.00	2.60
β-Conglycinin, mg/g	47.10	6.28
Small peptide, %	0.70	15.82

### 2.2 Animals, diets, and experimental design

A total of 120 healthy 28 days-old weaned piglets (Duroc × Landrace × large) with an average initial body weight (BW) of 7.0 ± 0.4 kg were allotted to 4 dietary treatments in a randomized complete block design with 5 replicates per treatment of 6 piglets for each replicate. The control group was fed a basal diet, and the 3 experimental groups were fed diets supplemented with 2, 4 and 8% ESBM instead of equal amounts of SBM. The pretest lasted for 5 days, and the formal growth test lasted for 28 days.

All utensils and environments were disinfected prior to feeding trials, and the formulation of the nonpharmaceutical corn-soybean meal based diet met or exceeded requirements recommended by the National Research Council (NRC, 2012) for piglets (13) (Table 3). Piglets had *ad libitum* access to water and feed.

**Table 3:** Composition and nutrient levels of the experimental diets (air-dry basis, %).

Items	Control group	Addition amount of enzymolytic soybean meal (%)		
		2	4	8
**Ingredients, %**				
Corn	33.94	34.04	34.14	34.34
Soybean meal	11.80	9.70	7.60	3.40
Enzymolytic soybean meal	—	2.00	4.00	8.00
Extruded corn	20.00	20.00	20.00	20.00
Extruded soybean	8.00	8.00	8.00	8.00
Flour	5.00	5.00	5.00	5.00
Whey powder	10.00	10.00	10.00	10.00
Fish meal	5.00	5.00	5.00	5.00
Glucose	2.00	2.00	2.00	2.00
Dicalcium phosphate	0.75	0.75	0.75	0.75
Limestone	0.50	0.50	0.50	0.50
Sodium chloride	0.50	0.50	0.50	0.50
L-lysine∙HCl	0.55	0.55	0.55	0.55
DL-methionine	0.14	0.14	0.14	0.14
L-threonine	0.16	0.16	0.16	0.16
L-valine	0.11	0.11	0.11	0.11
L-tryptophan	0.05	0.05	0.05	0.05
Premix^a^	1.50	1.50	1.50	1.50
**Nutrient levels** ^b^				
Net energy, kcal/kg	2,500	2,500	2,500	2,500
Crude protein	17.89	17.62	17.47	17.49
Ether extract	4.22	4.45	4.39	4.12
Calcium	0.74	0.74	0.74	0.74
Total phosphorus	0.62	0.62	0.62	0.62

a The Premix provides the following (per kg of diets): VA 12,000 IU, VD3 2,000 IU, VE 24 mg, VK3 3.5 mg, VB1 2.0 mg, VB2 6.0 mg, VB6 3.0 mg, VB12 24 μg, nicotinic acid 30 mg, D-pantothenic acid 20 mg, folic acid 3.6 mg, biotin 0.1 mg, choline chloride 400 mg, Fe (as ferrous sulfate) 96 mg, Cu (as copper sulfate) 8.0 mg, Zn (as zinc sulfate) 120 mg, Mn (as manganese sulfate) 40 mg, I (as calcium iodate) 0.56 mg, Se (as sodium selenite) 0.4 mg.

b Net energy, calcium and total phosphorus were calculated values, while the others were measured values. Net energy value was referred to Chinese National Feed Database of Swine (GB/T 39235-2020).

### 2.3 Growth performance and diarrhea incidence

At the start and end of the trail, the BW of piglets and feed disappearance were recorded to calculate the average daily gain (ADG), average daily feed intake (ADFI), and feed-to-gain ratio (F/G). According to the method described by Pan et al. (14), the fecal score was measured daily based on the clinical symptoms. 1 = hard feces, 2 = slightly soft feces, 3 = partially soft and formed feces, 4 = loose and semiliquid feces, 5 = watery feces. Fecal scores of 2 or 3 for 2 consecutive days were considered as the occurrence of diarrhea.

### 2.4 Samples collection and measurements

According to the Association of Analytical Communities (AOAC) (15), experimental diets were sampled for chemical analysis of dry matter (DM), gross energy (GE), crude protein (CP) and ether extract (EE). At the end of the experiment, one piglet with the average BW from each pen was chosen and euthanized for sample collection. Blood samples were collected from anterior cava vein into vacuum tubes without anticoagulant, and were then centrifuged at 3,500 × g for 10 min at 4°C, serum samples were collected and stored at −20°C until analysis. The middle section of duodenum, jejunum and ileum tissues were collected and fixed in 4% paraformaldehyde for intestinal morphology analysis. Jejunum tissue and cecal digesta were collected, rapidly frozen in liquid nitrogen, and stored at −80°C until further analysis. From day 25 to day 28, fresh fecal samples were collected, and dried in a forced air oven (60°C) for 72 h, grounded through a 1 mm screen for analysis of the apparent total tract digestibility (ATTD) of DM, GE, CP and EE using acid-insoluble ash (AIA) as a natural marker.

### 2.5 Intestinal morphology

Standard paraffin embedding procedures were used to dehydrate, extract, and embed fixed intestine specimens that had been kept in 4% paraformaldehyde. The sample was placed on a slide after being thinned to 5 mm. Following dewaxing, hematoxylin and eosin staining were performed on the paraffin slices. An image processing and analysis system (BA210Digital, Motic, China) was used to measure the villus height, crypt depth, and intestinal wall thickness under a microscope at a total combined magnification of 40 times. The ratio of villus height to crypt depth was calculated using the measurements of at least 10 intact, well-oriented villi and the related crypt depth for each section.

### 2.6 Antioxidant and immune indexes

The activities of total antioxidant capacity (T-AOC), glutathione peroxidase (GSH-Px) and total superoxide dismutase (T-SOD), as well as the concentrations of malondialdehyde (MDA), interleukin-8 (IL-8), interleukin-6 (IL-6), tumor necrosis factor (TNF-α), interferon-gamma (IFN-γ), lysozyme, D-lactate (D-LA) and diamine oxidase (DAO) in serum were analyzed using corresponding ELISA kit (Beijing Bori Long Range Technology Co., Ltd.).

### 2.7 Cecal microbiota

Approximately 5 mL of cecal digesta was collected in sterile cryopreservation tubes and stored at −80°C. Bacterial genomic DNA from cecal digesta was extracted using a fecal DNA kit (Omega Bio-Tek Incorporated, Norcross, United States). The V3–V4 region of bacterial 16S rRNA genes was amplified using the forward primer 338F (5′-ACTCCTACGGGAGGCAGCA-3′) and the reverse primer 806R (5′-GGACTACHVGGGTWTCTAAT-3′). Pair-end 2,250 bp sequencing was performed using the Illumina NovaSeq platform with the NovaSeq 6000 SP Reagent Kit (500 cycles) at Shanghai Personal Biotechnology Co., Ltd. (Shanghai, China). Sequence data were mainly analyzed using QIIME2 (2023.8) and the R package (v3.2.0). ASV tables in QIIME2 were used to calculate alpha diversity indices, such as Chao1 richness estimates, Shannon diversity and Simpson indices, and were presented as box plots. The principal coordinate analysis (PCoA) was performed using the PCoA tool in R language. Taxonomy compositions and abundances were visualized using MEGAN and GraPhlAn. The linear discriminant analysis effect size (LEfSe) was performed to detect differentially abundant taxa across groups based on Kruskal–Wallis and Wilcoxon tests. The original contributions presented in the study are publicly available. This data can be found here: NCBI SRA database (sequence number: PRJNA1073716).

### 2.8 Quantitative reverse transcription-PCR

The jejunum was separated from piglets after slaughter, frozen in liquid nitrogen, and stored in a −80°C freezer for testing. Total RNA was extracted from jejunum with an RNAiso Plus kit (TaKaRa, China), and the RNA concentration was detected by an ultrafine spectrophotometer. The quality of RNA was determined by electrophoresis apparatus (Mini Pro 300 V Power Supply, major science, United States). Small molecular bands of 28 s, 18 s and 5 s were observed after electrophoresis, indicating good integrity of the RNA. The cDNA was synthesized with HiScript III RT SuperMix of qPCR kit and stored at −20°C. The gene was amplified using reverse-transcribed cDNA as a template and detected by real-time fluorescent quantitative PCR (Vazyme Biotech). The reaction procedure was as follows: 95°C 30 s → 95°C 10 s → 60°C 30 s (40 cycles); melting curve 95°C 15 s → 60°C 60 s → 95°C 15 s. Glyceraldehyde-3-phosphate dehydrogenase (GAPDH) was used as the internal control, and the relative mRNA expression levels of the genes in each group were calculated by 2^−ΔΔCT^. The primers used in this study are shown in Table 4. The mRNA sequence was obtained from the National Center for Biotechnology Information (NCBI).

**Table 4:** Sequence of primers for real-time PCR.

Target gene	Primer sequence (5′to 3′)	GenBank accession number
ZO-1	Forward: CAAGAGAAGAACCAGATA	ENSSSCG00040044113
	Reverse: GTATGAAGGCGAATAATG	
Occludin	Forward: TCAGGGTGCACCCTCCAGATT	NM_001163647
	Reverse: ATGTC CGTTGCTGGGTGCATA	
Claudin-1	Forward: ACCCCAGTCAATGCCAGATA	ENSSSCT00060047372.1
	Reverse: GCGAAGGTTTTGGATAGGGC	
SOD1	Forward: GAGAAGACAGTGTTAGTAA	ENSSSCG00035056538
	Reverse: CAGCCTTGTGTATTATCT	
GSH-Px	Forward: GTGCTGCTCATTGAGAACGT	ENSSSCG00000060656
	Reverse: TCGGACGTACTTGAGGCAAT	
CAT	Forward: CAACAGTGCCAACGAAGA	ENSSSCG00070003335
	Reverse: TTCCTCTCCTCCTCATTCAG	
Nrf2	Forward: GCCCAGTCTTCATTGCTCCT	NM_001163647
	Reverse: AGCTCCTCCCAAACTTGCTC	
GAPDH	Forward: TCTGGCAAAGTGGACATT	ENSSSCG00035081064
	Reverse: GGTGGAATCATACTGGAACA	

ZO-1, zona occludens 1; SOD1, superoxide dismutase 1; GSH-Px, glutathione peroxidase; Nrf2, nuclear factor; CAT, catalase; GAPDH, glyceraldehyde-3-phosphate dehydrogenase.

### 2.9 Analysis of cecal fermentation characteristics

The cecal digesta was collected and temporarily stored in liquid nitrogen, and then stored at −80°C for the analysis of cecal fermentation characteristics. 0.5 g of digesta and 2.5 mL of pre-cooled ultra-pure water were mixed using a vortex oscillator. After 10 min of centrifugation at 4°C (12,000 rpm), the supernatant was collected. 25% metaphosphate was added to the solution of supernatant at the ratio of 5:1, acidified in an ice bath for 40 min, and centrifuged at 4°C (12,000 rpm) for 10 min. The contents of acetic acid, propionate, isobutyrate, butyrate, isovalerate and valerate in cecal digesta were determined by a gas mass spectrometer (GC2014, Shimadzu Inc., Japan). The concentrations of NH_3_-N and biogenic amines (tryptamine, putrescine, cadaverine, spermidine, and histamine) were analyzed by high-performance liquid chromatography (U3000, Thermo, United States). The Cecal digesta (0.5 g) was mixed with 1 mL of trichloroacetic acid and centrifuged at 3,600 × g for 10 min. The supernatant was mixed with the same volume of n-hexane and swirled for 5 min. The extract was mixed with 20 mL of internal standard, and then 1 mL of sodium hydroxide, 1 mL of dansyl chloride and 1.5 mL of saturated sodium bicarbonate were added. The mixture was heated at 60°C for 45 min, occasionally shaken, mixed with 100 mL ammonia and kept in a water bath at 40°C under nitrogen conditions. Finally, an analytical sample was prepared by adding acetonitrile to the residue. The wavelength, flow rate and column temperature were set at 254 nm, 1.0 mL/min and 40°C, respectively.

### 2.10 Statistical analysis

Statistical analyses were performed with SPSS 26.0 software. Results were presented as the means and standard error of the mean (SEM). Data were subjected to one-way ANOVA followed by Turkey’s multiple-range tests. Linear and quadratic comparisons were used to determine the effect of increasing ESBM. Significant differences were defined as *p* < 0.05, and tendencies were defined as 0.05 ≤ *p* < 0.10.

## 3 Results

### 3.1 Growth performance and diarrhea rate

Results showed that replacing SBM with 4% ESBM in the diet increased the ADG of weaned piglets (*p* < 0.05, Table 5). Compared with the control group, the diarrhea rate of piglets decreased linearly with the increasing addition of ESBM (*p* < 0.05). There were no differences in ADFI and F/G among the ESBM groups and the control group.

**Table 5:** Effects of enzymolytic soybean meal on the growth performance of weaned piglets.

Items^1^	Control group	Addition amount of enzymolytic soybean meal (%)			SEM	*p*-value		
		2	4	8		ANOVA	Linear	Quadratic
Initial BW (kg)	7.44	7.30	7.44	7.45	0.07	0.872	0.814	0.619
Final BW (kg)	14.76	14.87	15.60	15.06	0.13	0.087	0.132	0.180
ADFI (g)	426.95	478.59	460.3	421.18	16.87	0.614	0.822	0.211
ADG (g)	261.3^b^	270.37^ab^	291.37^a^	271.85^ab^	4.07	0.048	0.112	0.058
F/G	1.63	1.78	1.58	1.55	0.06	0.579	0.438	0.476
Diarrhea incidence (%)	4.29^a^	1.43^b^	1.67^b^	0.74^b^	0.39	0.001	0.001	0.092

^1^BW, body weight; ESBM, enzymolytic soybean meal; ADG, average daily gain; ADFI, average daily feed intake; F/G = feed intake/weight gain. ^2^Mean values differ significantly (*p* < 0.05) within the same row for different letters; SEM, standard error of mean. *n* = 5.

### 3.2 Nutrient digestibility

The ATTD of DM, GE and EE in piglets fed the 4% ESBM group were higher than those in other groups (*p* < 0.05). Moreover, A linear increase (*p* < 0.05) in the ATTD of CP was observed with increasing ESBM in the diets (Table 6).

**Table 6:** Effects of enzymolytic soybean meal on the apparent digestibility of nutrients in weaned piglets.

Items^1^	Control group	Addition amount of enzymolytic soybean meal (%)			SEM	*p*-value		
		2	4	8		ANOVA	Linear	Quadratic
DM	83.55^b^	82.67^b^	85.68^a^	82.00^b^	0.43	0.005	0.577	0.045
GE	82.68^b^	82.22^b^	84.85^a^	80.86^b^	0.45	0.006	0.364	0.020
CP	77.52	78.92	79.55	81.77	0.60	0.078	0.013	0.706
EE	64.86^b^	64.07^b^	71.00^a^	61.17^b^	1.22	0.019	0.644	0.036

^1^DM, dry matter; GE, gross energy; CP, crude protein; EE, ether extract. ^2^Mean values differ significantly (*p* < 0.05) within the same row for different letters; SEM, standard error of mean. *n* = 5.

### 3.3 Intestinal morphology

There was a tendency (linear, *p* = 0.078) for increasing villus height in the jejunum as dietary ESBM increased (Table 7). Likewise, the villus height in ileum of piglets linearly increased (*p* = 0.006) and the ratio of villus height to crypt depth tended to increase (linear, *p* = 0.088) with increasing ESBM in the diets. ESBM supplementation did not affect the intestinal morphology of duodenum and mucosal thickness of duodenum, jejunum and ileum.

**Table 7:** Effects of enzymolytic soybean meal on the intestinal morphology of weaned piglets.

Items^1^	Control group	Addition amount of enzymolytic soybean meal (%)			SEM	*p*-value		
		2	4	8		ANOVA	Linear	Quadratic
**Duodenum**								
VH (μm)	600.54	696.22	674.11	597.83	20.43	0.385	0.891	0.096
CD (μm)	349.31	378.90	460.84	382.88	17.30	0.119	0.241	0.108
V:C	1.71	1.93	1.49	1.56	0.08	0.246	0.247	0.629
MT (μm)	381.90	356.69	416.52	407.80	20.99	0.763	0.538	0.861
**Jejunum**								
VH (μm)	556.73	633.68	738.20	666.72	28.01	0.137	0.078	0.170
CD (μm)	283.36	348.70	336.88	279.13	16.12	0.309	0.864	0.068
V:C	1.99	1.97	2.25	2.42	0.13	0.594	0.209	0.735
MT (μm)	254.15	312.92	254.19	307.80	13.02	0.199	0.367	0.918
**Ileum**								
VH (μm)	341.13^c^	473.80^ab^	399.51^bc^	488.48^a^	18.09	0.003	0.006	0.412
CD (μm)	312.46	280.69	299.90	304.31	12.99	0.870	0.967	0.523
V:C	1.12	1.71	1.44	1.65	0.09	0.087	0.088	0.266
MT (μm)	325.58	401.69	363.76	386.35	11.57	0.093	0.139	0.216

^1^VH, Villus height; CD, crypt depth; V:C, Villus height: crypt depth; MT, mucosal thickness. ^2^Mean values differ significantly (*p* < 0.05) within the same row for different letters; SEM, standard error of mean. *n* = 5.

### 3.4 Determination of inflammatory cytokines and antioxidant activity

There was a quadratic increase (quadratic, *p* < 0.05) in the activity of SOD and T-AOC, and IFN-γ level in the serum of piglets with increasing dietary ESBM supplementation (Table 8). There were no differences in the activities of lysozyme and GSH-PX, and the concentrations of MDA, IL-6, IL-8 and TNF-α in serum among treatments. The addition of ESBM linearly increased (*p* < 0.05) the activity of LYS.

**Table 8:** Effects of dietary enzymolytic soybean meal supplementation on serum cytokines contents and antioxidant capacity of piglets.

Items^1^	Control group	Addition amount of enzymolytic soybean meal (%)			SEM	*p*-value		
		2	4	8		ANOVA	Linear	Quadratic
SOD (U/mL)	309.57^c^	404.65^a^	370.30^ab^	344.51^bc^	11.39	0.006	0.430	0.003
MDA (nmol/mL)	8.25	8.01	7.64	7.50	0.24	0.719	0.284	0.923
GSH-Px (U/mL)	572.38	580.14	624.46	643.50	19.76	0.522	0.146	0.889
T-AOC (U/mL)	31.24^b^	38.49^a^	37.16^a^	35.88^a^	0.88	0.007	0.102	0.003
IL-8 (pg/mL)	284.15	293.24	293.67	278.09	6.46	0.823	0.776	0.381
IL-6 (pg/mL)	1059.77	1144.04	1189.64	1052.53	28.87	0.274	0.925	0.065
TNF-α (pg/mL)	1275.64	1279.25	1298.56	1228.56	23.49	0.761	0.561	0.457
IFN-γ (pg/mL)	59.40	64.68	65.78	61.44	1.18	0.196	0.494	0.044
LYS (U/L)	9.21	11.80	10.75	14.90	1.05	0.071	0.027	0.602

^1^MDA, malondialdehyde; T-AOC, total antioxidant capacity; SOD, superoxide dismutase; GSH-Px, glutathione peroxidase; IL, interleukin; TNF-α, tumor necrosis factor-α; IFN-γ, interferon-γ; LYS, lysozyme. ^2^Mean values differ significantly (*p* < 0.05) within the same row for different letters; SEM, standard error of mean. *n* = 5.

### 3.5 Intestinal permeability

**Figure 1:**
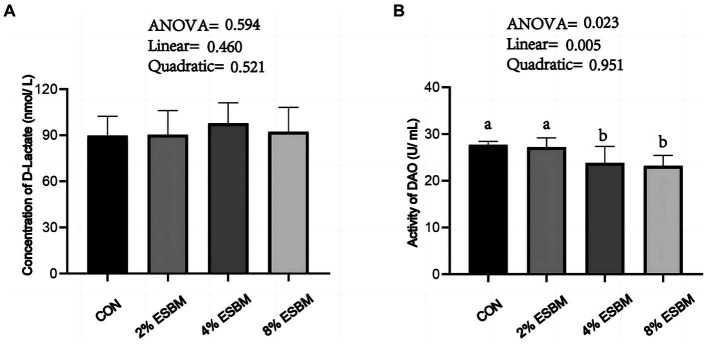
Effect of enzymolytic soybean meal on intestinal permeability of piglets. **(A)** D-lactate **(B)** DAO, diamine oxidase. No letter above the bar or the same letter indicates no significant (*p* > 0.05, *n* = 5), while with different letter mean significant difference (*p* < 0.05).

The concentration of D-lactate in ESBM groups had no difference when compared with the control group (Figure 1A), the DAO content decreased linearly (*p* < 0.05) with the increasing addition of ESBM (Figure 1B).

### 3.6 Cecal microbiota

**Figure 2:**
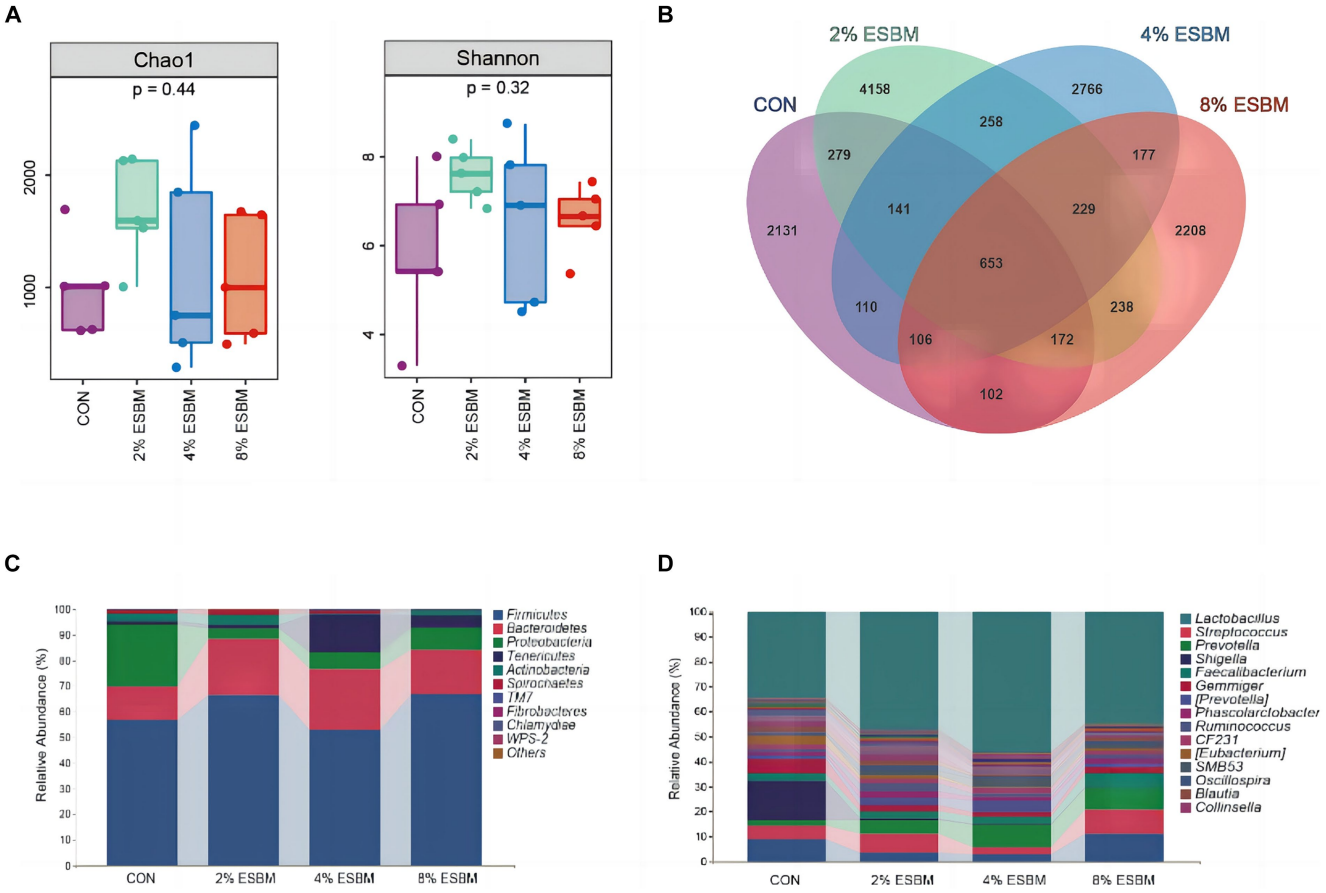
Cecal microbiota diversity and richness. **(A)** Comparison of a-diversity indices among 4 dietary treatments. **(B)** OTU Venn diagram of the 4 dietary treatments. Relative abundance of cecal microbiota at the **(C)** phylum and **(D)** genus levels.

**Figure 3:**
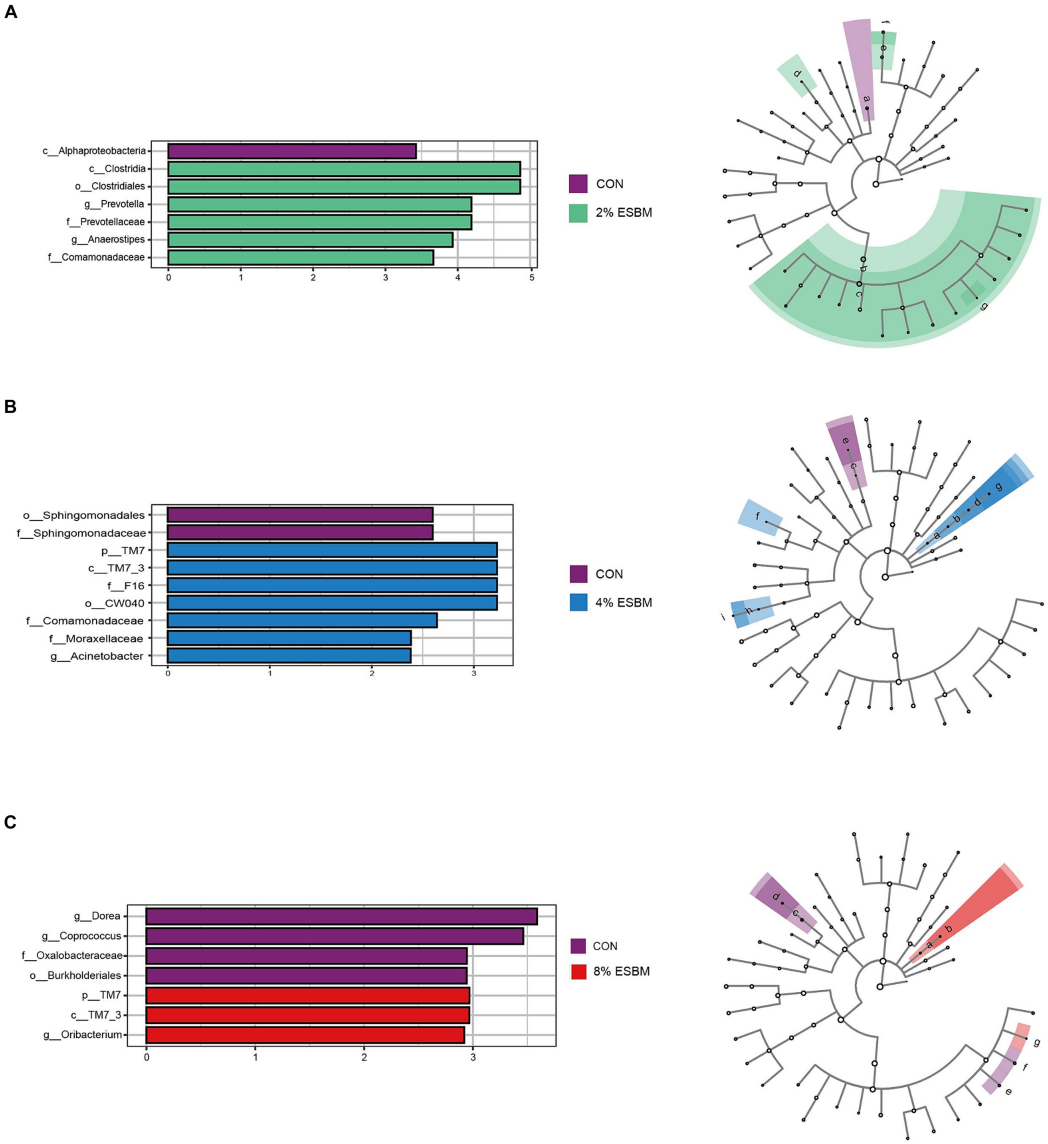
LEfSe analysis and LDA score distribution histogram of effects of ESBM on cecal microbial communities of piglets, the current LDA threshold is 2.

The Shannon index and Chao1 index in cecal samples in the ESBM groups did not differ from the control group (Figure 2A). Among the four diets, there were 2,131, 4,158, 2,766, and 2,208 differential OTUs identified in cecal digesta (Figure 2B). Firmicutes and Bacteroidetes accounted for 80% of all bacteria at the phylum level (Figure 2C). At the genus level, the dominant genera within Firmicutes included *Lactobacillus*, *Faecalibacterium* and *Phascolarctobacterium*, and *Prevotella* was the dominant genus of Bacteroides (Figure 2D). To confirm the differences in bacteria between taxa, LEfSe was performed on all levels of taxa to find the characteristic genera between taxa (Figure 3). *g_Prevotella* and *g_Anaerostipes* were specific bacteria in the 2% ESBM group (Figure 3A); *g_Acinetobacter* was a specific bacterium in the 4% ESBM group (Figure 3B). *g_Oribacterium* was a special bacterium in the 8% ESBM group (Figure 3C).

### 3.7 Expression of antioxidant and intestinal barrier genes in the jejunum

**Figure 4:**
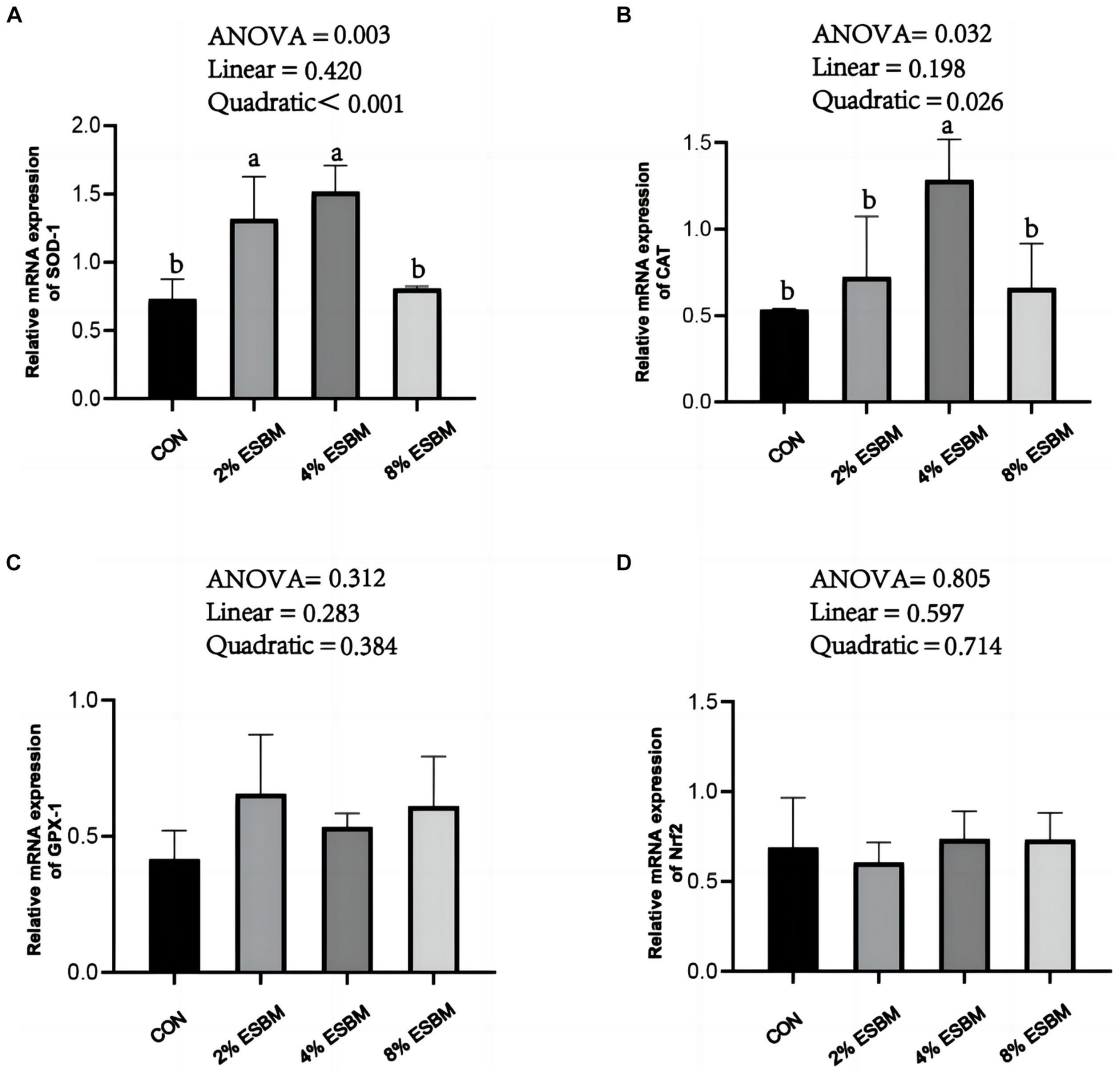
**(A)** SOD-1 **(B)** CAT **(C)** GPX-1 **(D)** Nrf2. Effect of enzymolytic soybean meal on the expression of antioxidant genes in the jejunum of piglets. No letter above the bar or the same letter indicates no significant (*p* > 0.05, *n* = 5), while with different letter mean significant difference (*p* < 0.05).

**Figure 5:**
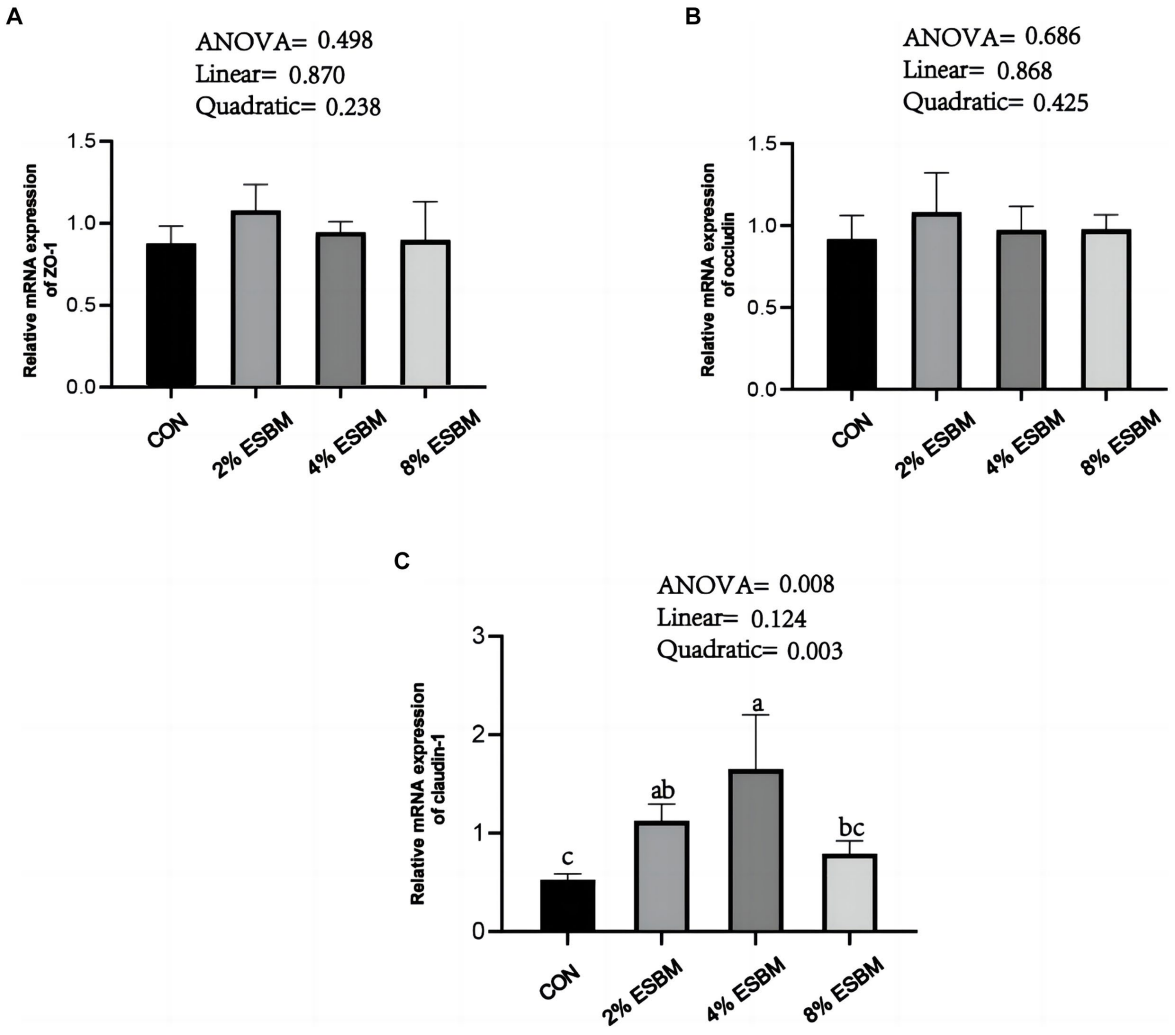
**(A)** ZO-1 **(B)** Occludin **(C)** Claudin-1. Effect of enzymolytic soybean meal on intestinal barrier gene expression in jejunum of piglets. No letter above the bar or the same letter indicates no significant (*p* > 0.05, *n* = 5), while with different letter mean significant difference (*p* < 0.05).

The mRNA expression of *SOD1* and *CAT* in jejunum of piglets increased quadratically (*p* < 0.05) as dietary ESBM increased (Figures 4A,B). As well as, compared with control group, the 2 and 4% ESBM groups increased (*p* < 0.05) the mRNA expression of *SOD1* (Figure 4A), and the 4% ESBM group increased (*p* < 0.05) the mRNA expression of *CAT* (Figure 4B). The mRNA expression of *GPX-1* and *Nrf2* in the jejunum of piglets was not affected by the addition of ESBM (Figures 4C,D). As shown in Figure 5, compared with the control group, the mRNA expression of *ZO-1* and *occludin* in jejunum of ESBM group showed no significant difference (Figures 5A,B). The addition of ESBM quadratically increased (*p* < 0.05) the mRNA expression of *claudin-1* in jejunum (Figure 5C).

### 3.8 Short-chain fatty acids

Compared with the control group, feeding ESBM had a tendency (*p* = 0.073) to increase the concentration of butyrate, and the content of valerate increased quadratically (*p* < 0.05) with increasing addition of ESBM (Table 9). In addition, ESBM addition linearly increased (*p* < 0.05) the concentrations of acetate, propionate and total short-chain fatty acids (SCFAs).

**Table 9:** Effects of enzymolytic soybean meal on short-chain fatty acids in the cecal digesta of weaned piglets (mmol/L).

Items	Control group	Addition amount of enzymolytic soybean meal (%)			SEM	*p*-value		
		2	4	8		ANOVA	Linear	Quadratic
Acetate	35.70	45.26	48.80	48.02	2.42	0.193	0.060	0.275
Propionate	13.61	16.73	16.83	19.92	0.96	0.138	0.029	0.996
Butyrate	3.77	7.27	5.38	8.35	0.69	0.073	0.044	0.828
Valerate	0.39^b^	1.06^a^	0.98^a^	0.97^a^	0.09	0.026	0.027	0.043
Isobutyrate	0.17	0.23	0.37	0.30	0.04	0.262	0.123	0.343
Isovalerate	0.11	0.15	0.25	0.20	0.16	0.510	0.244	0.556
Total SCFAs	53.70	70.69	74.57	77.77	3.88	0.163	0.046	0.597

Mean values differ significantly (*p* < 0.05) within the same row for different letters; SEM, standard error of mean. *n* = 5.

### 3.9 Cecal fermentation characteristics

The tryptamine concentration decreased (linear, *p* < 0.05; quadratic, *p* < 0.05) with increasing addition of ESBM (Table 10). Moreover, the content of cadaverine and NH_3_-N presented a quadratic decrease (*p* < 0.05), and histamine presented quadratic increase (*p* < 0.05) as increasing addition of ESBM.

**Table 10:** Effects of enzymolytic soybean meal on NH_3_-N and bioamine concentrations in cecal digesta of piglet (mg/kg).

Items	Control group	Addition amount of enzymolytic soybean meal (%)			SEM	*p*-value		
		2	4	8		ANOVA	Linear	Quadratic
Tryptamine	1.24^a^	0.60^b^	0.55^b^	0.83^b^	0.06	0.010	0.036	0.004
Putrescine	28.50	33.96	22.28	33.72	1.86	0.055	0.758	0.314
Cadaverine	8.89^ab^	7.25^b^	6.66^b^	12.12^a^	0.80	0.034	0.107	0.013
Histamine	8.97	7.72	7.33	11.26	0.66	0.131	0.231	0.049
Spermidine	79.77	66.99	70.10	60.79	3.59	0.333	0.118	0.807
Spermine	13.90	20.67	14.57	12.68	1.90	0.511	0.592	0.299
NH_3_-N	40.96^a^	37.98^a^	30.10^b^	40.93^a^	1.02	0.001	0.269	0.001

Mean values differ significantly (*p* < 0.05) within the same row for different letters; SEM, standard error of mean. *n* = 5.

## 4 Discussion

Enzymatic hydrolysis can reduce the anti-nutritional factors of SBM, and decompose the protein, sugar and other organic matter into small molecules to improve the nutritional value, so that it is easier to be digested and absorbed (8, 16). The protein structure may affect the amino acid (AA) absorption kinetics due to enzyme affinity and transporter specificity (17). Prior to this study, we compared the AA content of ESBM and SBM, and the results showed that the free AA content of ESBM was higher than that of SBM. Unlike intact proteins, free AA can be rapidly absorbed and improve piglet digestion (18, 19). Furthermore, we found that the content of anti-nutritional factors (glycine and conglycinin) in ESBM was significantly lower than that in SBM. This is probably because the fraction α’ of β-conglycinin and the acidic fraction (AS) of glycinin were more susceptible to hydrolysis by protease in ESBM. Therefore, ESBM is a high-quality plant protein source. In this study, we discovered that the ADG of piglets receiving diets containing 4% ESBM was higher than that of control group, which was similar to the results of previous studies (4, 20), where 7.5–15.10% ESBM had greater ADG than those pigs fed soy protein concentrate (SPC) and fermented soybean meal (FSBM) diet. Additionally, we observed that a linear decrease in diarrhea incidence as the supplementation levels of ESBM increased. This finding was consistent with previous study (21), where found that feeding 7, 14% or 21% of ESBM diets improved overall fecal score. No appreciable variations in the ADFI and F/G of piglets fed ESBM were discovered. In this study, increasing addition of ESBM increased ATTD of DM, GE, and EE, which may contributed to the growth improvement in weaned piglets.

Intestinal morphology reflects the ability for intestinal digestion and absorption as well as intestinal health (22, 23). Since the small intestinal villi are a crucial site for nutrient absorption, piglets typically experience significant changes in intestinal structure and function after weaning. These changes are primarily characterized by villi atrophy and crypt hyperplasia, which reduces the ability to absorb nutrients (24). Therefore, we speculated that after the enzymatic hydrolysis of SBM, intestinal oxidative stress should also be reduced due to the decrease in the content of anti-nutritional factors. Meanwhile, the enzymatic hydrolysis of SBM produced many small peptides and other active factors, which might promote intestinal development. A previous study indicated that supplementation with 0.5–1.5% ESBM did not improve the intestinal morphology in the jejunum of Rex rabbits (25). In this study, the addition of ESBM linearly increased the villus height and tended to increase the ratio of villus height to crypt depth in the ileum, which was different from the results of previous studies (21), possibly due to the different addition amount of ESBM.

The oxidative stress of piglets may be caused by weaning. In order to observe the physiological status of piglets in time, the activity of anti-oxidant enzymes usually is determined (26, 27). Weaning stress has been shown to reduce GSH-Px and SOD activity and raise MDA level in serum of piglet (28). SOD is considered to be the first line of defense against excessive oxidizing free radicals and catalyzes the transformation of superoxide free radicals into H_2_O_2_, which is decomposed into H_2_O and O_2_ by GSH-Px and CAT (29). In this study, ESBM supplementation increased the SOD activity in the serum of piglets, indicating that ESBM had a better effect on improving enzymatic antioxidant defense, which is consistent with previous study (20). T-AOC levels reflect nonenzymatic antioxidant defense systems (30). Our study showed that increased ESBM in diets increased T-AOC levels, suggesting that ESBM plays an important role in preventing endogenous lipid peroxidation and oxidation. The response of antioxidant enzymes to exogenous and endogenous factors that cause oxidative stress depends on a number of factors, including the expression of genes that code for these enzymes (31). This study found that supplementing ESBM increased the expression levels of *SOD1* and *CAT* mRNA in the jejunum of piglets. The Nrf2 pathway is regarded as a primary cellular defense mechanism against oxidative stress (32). In this study, we found that supplementing ESBM had no effect on the expression of *Nrf2* in jejunum, which suggested that the anti-oxidative activities of ESBM may not be mediated by the regulation of *Nrf2*. The potential mechanism of ESBM to improve the antioxidant capacity of piglets needs further study. In addition, weaning stress increased the amount of *E. coli* and enterotoxins in piglet intestine, which led to inflammation by decreasing pro-inflammatory cytokines such as IL-6, IL-8, IFN-γ, and TNF-α (33, 34). In this study, no significant differences were observed on IL-6, IL-8, IFN-γ, and TNF-α levels among all groups, suggesting that ESBM supplementation had no significant effect on inflammatory response of weaned piglets. Lysozyme activity is one of the factors that trigger non-specific immunity, which has the characteristics of fighting harmful bacteria and reducing inflammation, and is closely related to animal health (35). In this study, the level of lysozyme increased linearly as the level of ESBM increased. A previous study reported that largemouth bass with enzymatically treated skinless soybean meal had considerably higher serum lysozyme activity and better body immunity (36), which was consistent with our study. The above results suggested that feeding a moderate amount of ESBM improved the serum antioxidant capacity of piglets.

To investigate the effects of ESBM on intestinal health of piglets, we detected the intestinal permeability and intestinal barrier function. Intestinal barrier integrity is essential for epithelial cells to function normally and prevent pathogenic microorganisms that cause inflammation from entering the body (37). The damage of intestinal barrier function increases the permeability of epithelial cells. In mammalian intestinal villous epithelial cells, DAO is a marker enzyme. Its activity is closely correlated with villous height and nucleic acid and protein synthesis in mucosal cells, and it can be used as a good indicator of impairment in intestinal mucosal barrier function (38, 39). The intestinal lumen contains D-lactate, which is a byproduct of bacteria (40). D-lactate can be utilized as an indicator of increased intestinal wall permeability because the intact intestinal mucosa serves as a barrier to stop D-lactate from reaching portal venous circulation (41). In this study, ESBM supplementation linearly reduced DAO content, while having no significant effect on D-lactate, which was similar to a previous study (42), suggesting that ESBM contributed to improve the integrity of the intestinal barrier. Intestinal barrier function is significantly influenced by tight junctions. Different junction molecules, including *ZO-1*, *Occludin*, and *Claudin*, make up tight junction in intestinal epithelial cells. These molecules control the paracellular permeability of water ions and macromolecules in neighboring cells (43). *Claudin1* is an efficient barrier against the invasion of infections, toxins, and antigens. In this study, ESBM boosted *Claudin1* expression in the jejunum of piglets. These improvements in *Claudin1* mRNA abundance may due to the reduced concentration of glycinin and β-conglycinin in ESBM (44). However, ESBM presented no impact on the mRNA expression of *Occludin* and *ZO-1*. Similar to our findings, researchers previously discovered that addition of 21% ESBM had no significant effect on mRNA expression of *ZO-1* and *Occludin* (21).

The microbiome is a complex ecosystem that has a significant impact on animal gut health (45). Mammals’ metabolic, nutritional, and physiological functions are also significantly influenced by the microbiome (46). Diversity, encompassing species richness (observed species, Chao and Ace) and species diversity (covered by Shannon, Simpson and Good), can be utilized as an indicator of the functional robustness of gut microbial ecosystems (47). In this study, cecal digesta from piglets fed various amounts of ESBM showed no change in species richness or species diversity. Similar to the previous study (25), ESBM did not significantly affect relevant indicators of cecal alpha diversity in Rex rabbits. We found that at the phylum level, the cecal flora of weaned piglets primarily consisted of Firmicutes and Bacteroidetes. Firmicutes are associated with the decomposition of polysaccharides and the utilization of energy in the gut due to their ability to encode genes for non-starch polysaccharide-degrading enzymes, bacteroides is a probiotic rich in carbohydrate metabolic pathways and polysaccharides degrading enzymes that break down nutrients in food for use by the animals’ organism (48). In this study, ESBM enhanced the abundance of *Provotella* in Bacteroides and *Oribacterium* in Actinomyces in cecal digesta. However, in terms of cecal microbiota composition, there was no discernible difference between the ESBM and control groups.

Normally, the foregut is where dietary nutrients are digested and absorbed, while the hindgut is where the gut bacteria ferment undigested components and endogenous chemicals. Due to the production of SCFAs, carbohydrate fermentation is advantageous to intestinal epithelial cells, whereas protein fermentation produces potentially toxic metabolites such as ammonia, bioamines, and aromatic compounds, which can adversely affect the intestine (49). In this study, valerate and butyrate contents increased linearly as ESBM increased. Butyrate is thought to be a significant source of energy for colon cells and can encourage the growth and differentiation of the intestinal epithelium (50). Ruckman et al. (21) showed that the concentrations of acetate, butyrate, propionate and total SCFA in ileal digestives increased linearly with increasing dietary ESBM content, which was consistent with the results in this study. Raffinose oligosaccharides in beans produce flatulence in man and animals, since animals lack the enzymes to hydrolyze these oligosaccharides, which are fermented by bacteria in hindgut to produce gas (51). NH_3_-N, a hazardous byproduct of amino acid deamination, can disrupt epithelial cell metabolic processes and damage the intestinal health of pigs (52, 53). Our findings demonstrated a considerable decrease in NH_3_-N in cecal digesta of piglets fed 4% ESBM compared to the control group, which further supported the higher growth performance of piglets fed 4% ESBM, and then ESBM tended to reduce tryptamine and cadaverine levels in cecal digesta indicating decreased protein fermentation, which may clarify the underlying mechanism in regulating gut health of dietary protein. In addition, this study confirmed that the contents of raffinose, hyperthyreose, sucrose and other oligosaccharides in ESBM were significantly reduced compared with SBM, indicating that ESBM reduced the production of harmful gases, which again confirmed the reliability of ESBM in inhibiting the production of fermentation gases in the cecum of piglets.

## 5 Conclusion

The substitution of SBM with ESBM resulted in growth improvement in weaned piglets, attributed to enhanced nutrient digestibility, antioxidant capacity, and intestinal barrier function. ESBM supplementation notably hindered protein fermentation in the hindgut. Our findings highlight the efficacy of 4% ESBM supplementation within the piglet diet under the experimental conditions. These results underscore ESBM’s potential as a crucial dietary component for enhancing piglet health and growth, providing valuable insights into swine nutrition optimization.

## Data availability statement

The original contributions presented in the study are publicly available. This data can be found at: https://www.ncbi.nlm.nih.gov/bioproject/; PRJNA1073716.

## Ethics statement

The animal study and the experimental protocols were approved by the Animal Care and Use Committee of Hebei Agriculture University (Baoding, China). All animal experiments complied with the ARRIVE guidelines and were carried out in accordance with the U.K. Animals (Scientific Procedures) Act, 1986 and associated guidelines, EU Directive 2010/63/EU for animal experiments. The study was conducted in accordance with the local legislation and institutional requirements.

## Author contributions

KT: Data curation, Methodology, Writing – original draft. ZB: Investigation, Supervision, Writing – review & editing. HL: Investigation, Software, Writing – review & editing. WH: Investigation, Methodology, Writing – review & editing. MX: Formal analysis, Methodology, Writing – review & editing. SH: Funding acquisition, Supervision, Writing – original draft. BC: Funding acquisition, Project administration, Resources, Supervision, Writing – original draft, Writing – review & editing.
